# Three-Dimensional Bone Block Planning for Mandibular Sagittal Bone Defect Reconstruction

**DOI:** 10.1155/2020/8829288

**Published:** 2020-10-05

**Authors:** M. Dominiak, S. Dominiak, S. Targonska, T. Gedrange

**Affiliations:** ^1^Department of Oral Surgery, Medical University of Wroclaw, Wroclaw, Poland; ^2^Institute of Low Temperature and Structure Research, Polish Academy of Sciences, Wroclaw, Poland; ^3^Department of Orthodontic, TU Dresden, Dresden, Germany

## Abstract

Bone defects seen in severe sagittal discrepancies between the maxilla and mandible do not solely qualify for orthodontic treatment. An interdisciplinary approach with the aid of a surgical, orthodontic, and periodontal team should be implemented in the treatment of such cases. Despite the use of standard treatment methods, the therapy is always planned and carried out individually for each patient. The surgical treatment of bone defects in the area of the jawbones is associated with a number of potential complications. Regenerative medicine, which has already been practiced in reconstructive surgery, is now gradually receiving more attention in the treatment of orthognathic defects. We developed a method for the reconstruction of the alveolar bone in the sagittal dimension using 3D allogenic graft blocks, as a preparing feature in the orthodontic treatment of borderline cases or as a treatment option for complications arising during general orthodontic treatment.

## 1. Technical Difficulties

**Figure 1:**
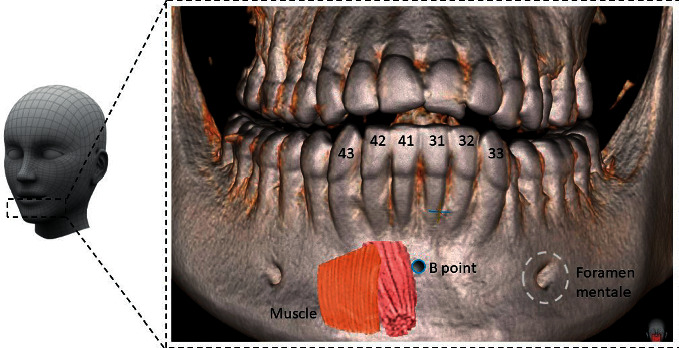
Anatomical structures of lower jaw.

The bone defects seen in the alveolar ridge of the maxilla/mandible are most commonly the result of various congenital genetically modified processes like hypodontia and/or due to acquired causes like odontogenic inflammatory processes, injuries, or tumors [1–3]. Regarding the morphological causes for bone defects, the most important one is the location, particularly the area of the mandibular lower incisors [4]. It has been stated that orthodontic movements in lingual or labial directions in this area are a cause of gingival recession [5–7]. Nevertheless, gingival recession is most commonly described in patients with insufficient bone width and bone height on the lingual and/or labial side of the mandibular alveolar bone [8]. Hence, tooth movement in the mandible is bound to certain anatomical restrictions (Figure 1). In case of decreased external bone substance, a risk of crossing these borders emerges which may lead to bone defects, recessions and—in the worst case scenario—tooth/teeth loss with bone loss of the surrounding alveolar ridge [8, 9]. Regardless of a possible cause, several methods of reconstruction exist [10].

When it comes to full dental arches, the main causes of bone defects are periapical inflammatory processes (intrabony defects) or marginal inflammations with periodontitis resulting in horizontal alveolar bone loss and vertical bone defects located mainly in the frontal or horizontal projection. For their reconstruction, different types of bone graft substitutes and osteogenic materials are used, delivering expected therapeutic effects. However, sagittal bone defects as a result of the alveolar ridge morphology (e.g., hypodontia and long and narrow mandibular symphysis) or morphological malocclusion (e.g., retrogenia and micrognathia), as well as the acquired sagittal bone defects as a consequence of orthodontic treatment, have not yet been successfully reconstructed [11]. Bone graft substitutes have already been used in attempt to rebuild bone volume on the vestibular side [12, 13]. The main advantages of this technique are its easy accessibility and procedural speed, while the disadvantages are low predictability due to insufficient reconstructed alveolar width and displacement of the material as a consequence of gravity and soft tissue tension. Furthermore, anatomical limitations such as the mental foramen need to be considered [14, 15]. However, the most important limitation is the quality of the newly formed bone. It is a conglomerate of biomaterial and bone without signs of vital bone tissue. This reconstructed conglomerate hinders an orthodontic treatment in this area due to its high density and the difficulty of moving teeth into this region [16, 17]. Moreover, the minor width of the reconstruction shows insufficiency to achieve a completely successful and effective orthodontic treatment or to be able to substitute orthognathic treatment procedures with general orthodontic treatment [18]. Especially in the case of borderline malocclusions where orthognathic treatment is recommended, but only orthodontic compensatory treatment is performed, difficulties will arise. Orthodontic compensatory treatment aims to correct the position of teeth in relation to the arch and occlusion, moving the teeth through the alveolar process. This treatment often depends on significant dental arch expansion which results in the movement of the teeth on the external surface of the alveolar process, creating bone dehiscences on one or both sides of the alveolar ridge. In severe ridge resorptions, the completion of an orthodontic treatment is not possible due to observed tooth mobility. To prevent this, a method for the reconstruction of the alveolar bone in the sagittal dimension using 3D allogenic bone blocks has been developed. This method is used as an element of preparing the patient for orthodontic treatment in borderline cases or as a treatment choice for complications during orthodontic treatment.

The purpose of this work is to introduce a three-dimensional reconstructive method for bone defects, using an individualized allogenic bone block.

## 2. How to Perform This Task?

### 2.1. Preparatory Phase

The correct design of the bone block is based on the analysis of skeletal and soft tissue parameters of the face and the alveolar process.

In the first stage, the block should be positioned in relation to the facial type (skeletal parameters).

**Figure 2:**
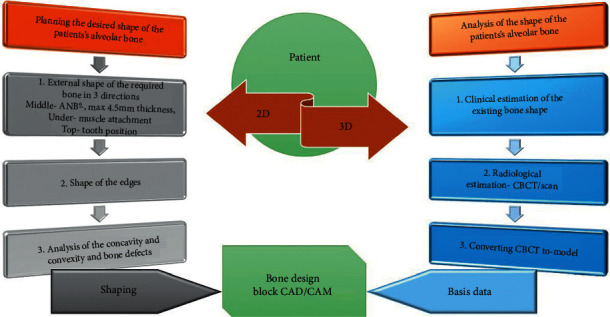
Diagnostics and planning of bone defect. Full 2D and 3D patient documentation shall be prepared.

For the positioning, basic orthodontic diagnostics as well as full 2D and 3D patient documentation should be prepared (Figure 2). Furthermore, to obtain individual 3D patient data, it is necessary to take a cone beam computed tomography (CBCT) scan along with intraoral and extraoral face scans and a lateral cephalogram. To obtain a valid cephalogram, a correct position of the condyles in centric relation needs to be assured during the X-ray procedure. During X-ray exposure, the patient should be positioned upright in the cephalostat, with their teeth in centric occlusion and the lips relaxed. Failure to comply with the principle of centric relation and the presence of double or forced occlusion may lead to an incorrect assessment of the type of malocclusion followed by improper treatment planning.

In the second stage, the positioning is based on the bone parameters of the alveolar process (analysis in 3 directions: height, width, and thickness including additional parameter, i.e., the shape of the internal surface of the block expressed by concavity or convexity of the alveolar ridge and/or bone defects).

In the third stage, muscle and soft tissue parameters are evaluated. It is important to evaluate the thickness of the mentalis muscle using clinical (“orange peel”) diagnosis and radiological (cephalometric analysis or CBCT scan) diagnostics or elastographic imaging [19, 20]. For the assessment of the soft tissue thickness and height, it is necessary to perform clinical and ultrasonic measurements [21–23].

#### 2.1.1. Documentation and Patient Diagnostics

**Figure 3:**
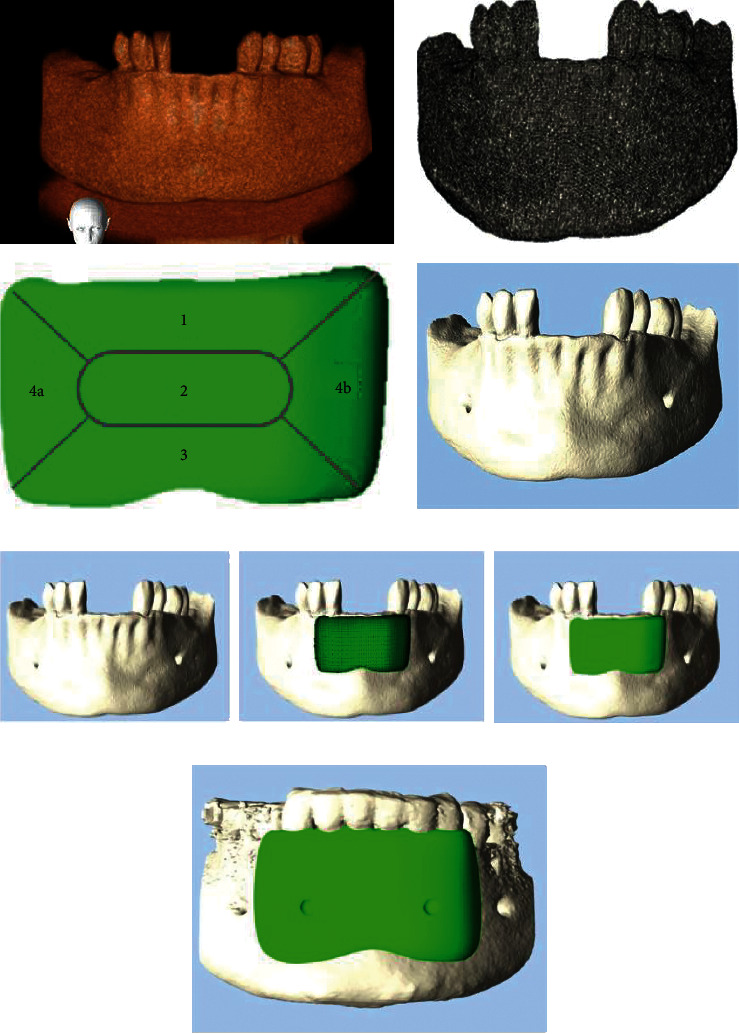
Planning of bone block and model of lower jaw. Defect planning divided into 4 external regions and 1 internal region. End planning from patient with positioning of screw for anchorage.

The entire area of the defect is divided into 4 external (Figure 3) and 1 internal regions. The divisions allow for a rough orientation so that further steps can be undertaken. First of all, reference points must be marked on the created recipient site. They determine the actual situation. A fully individualized bone block must be of high fitting accuracy.

Next, the internal surface of the implant is determined. Its contact area with the bone must be very wide so that cells can move from the bone to the implant and reconstitute there. The shape of the human mandible shows many individual anatomical variations which must be taken into account, i.e., mental nerve, mental protuberance, nerve canal, concavity, and convexity of the alveolar ridge. The latter is an important determining factor since it is formed by the position of the dental roots. A 3D X-ray image can visualize the variation in the course of the nerve canal. Additionally, the topography of the mandibular incisor region is based on many determining factors: shape and length of dental roots, structure and extent of the alveolar bone, nature of the covering soft tissue, periodontal clinical attachment level, and muscle insertion. This particular anatomy of the mandible allows the creation of a base for the implant.

**Figure 4:**
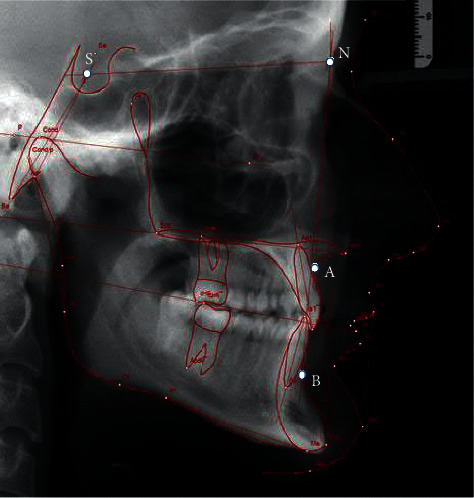
Cephalometric analysis with the use of lateral cephalograms.

External assessment is first carried out with the use of a cephalogram (Figure 4). Cephalometric analysis which relies upon the production of a lateral cephalogram provides valuable information on the shape, position, and relationship of the jaw bones in the vertical and sagittal plane. There are many analytical methods based on various measurements depending on angles and distances, as well as on various normative concepts [24–27]. The position of the jaw bones to each other and the position of the teeth must be presented and evaluated separately. Thereafter, the values can be evaluated and diagnosed. To predict the correct position of the anterior teeth, “floating” normative values based on the ANB angle were used, as described by Steiner [28]. Hasund [29–31] established an analysis for the entire cephalometry, which not only is based on average values but also describes how the respective values influence each other. The introduction of “floating” standards and leading variables facilitates the performance of individual cephalometric analysis for each patient [32].

Assessment of the ANB angle is applicable for the evaluation of the skeletal relationship between the maxilla and the mandible. In the sagittal plane, the ANB angle is used to describe bone changes (Figure 4). The ANB angle is classified by the differences between the SNA and SNB measurements and it depends on the facial type. According to Björk [33], there are three facial types: retrognathic, orthognathic, and prognathic. This classification is primarily based on the basic values of the sagittal angles (SNA and SNB). The optimum ANB angle for setting incisors is −1° for a retrognathic face, +2° for an orthognathic face, and +4° to +5° for a prognathic face. Changes in normative values for different ANB angles can be taken from the harmony box.

It should be underlined that the clinical significance of the ANB angle as a characterizing factor for malocclusions can only be defined in relation to the facial type.

When assessing the ANB angle, it is important to recognize the origin of the bone defect in order to allow the design of a targeted individual intervention.

For example, if the ANB angle is large, an error may occur in the mandible (mandibular retrognathism) or in the maxilla (maxillary prognathism).

The assessment of the actual mandibular incisor position is carried out prior to the treatment initiation, regarding two parameters. First, the correlation between the incisor or implant position and the mandible is analyzed. Then the NB line is used to cephalometrically determine the inclination of the mandibular incisors.

The relationship between the maxillary and mandibular incisors is largely influenced by the surrounding soft tissues and must be taken into account for treatment planning. The position of the incisors in relation to the tongue and lips is determined by the function of the soft tissues. A relatively balanced functional relationship of lips and tongue can be expected.

The actual position of the incisors describes the position of the teeth in the jaw as well as occlusion. If the position of the incisors in the mandible corresponds to the calculated target value, then the incisors are balanced between the lip and tongue. All parafunctions and impregnations of the tongue or lips must be specified in diagnostic documentation.

The distance between the upper incisal edge of the most anterior lower central incisor to the facial plane is determined by the Iis-NB ratio. The Iis-NB ratio and the position of mandibular incisors are the 4 mm distance between the incisal edge (Iis) of the mandibular central incisor and the NB reference line. The axial position of mandibular incisors in relation to the vertical line NB is 25°.

Alveolar bone level: a part from the position of the mandibular incisors, the alveolar ridge is to be measured [19, 34, 35].

**Figure 5:**
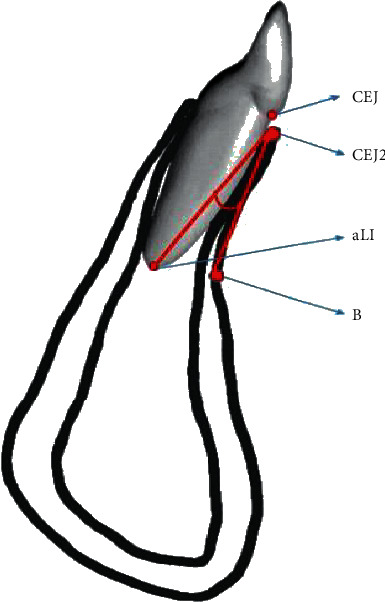
Measurement of the incisors position and alveolar bones.

An angle is formed based on the assessment of the point of deepest concavity on the anterior surface of the mandibular symphysis (point B) and the apical point of the most anterior mandibular central incisor (Iia) in relation to the cementoenamel junction of the incisor (CEJ) minus 2 mm which corresponds to the gingival sulcular depth (CEJ2). This evaluation method allows the prediction of bone resorption depending on tooth movement (Figure 5).

#### 2.1.2. Shape Planning of the Entire Bone Block and Position of the Dental Crowns

The actual reference point values are added to the target values. A new area, consisting of target values, is added to the diagnosed defect area. For the calculation, the mandibular segments with the applied bone block need to be analyzed in two planes. The images are obtained from the CBCT scans.

The first plane correlates with the bone block body (region 2). For this purpose, the ANB angle is used; after reconstruction this angle needs to match facial harmony. Special attention should be paid to the chin shape and the block's design should be individually adjusted. Hasund's cephalometric analysis provides the targeted values corresponding to the actual values.

The implant thickness should not exceed the possible physiological bone regeneration.

**Figure 6:**
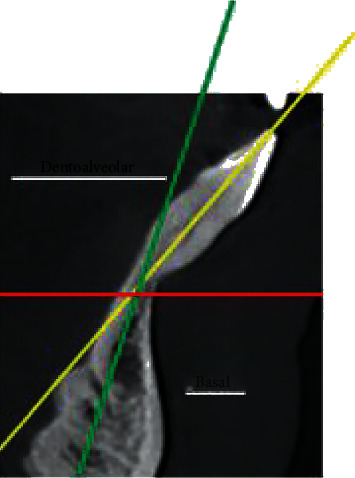
Long axes of incisor/alveolar process and basal symphysis.

For successful bone reconstruction, bone cells need to work in balance, an optimal blood circulation needs to be assured, and the value of the ANB angle should not exceed 4-5° after reconstruction. There are two additional conditions which determine the thickness of the bone block. One describes when the bone block is able to be thicker in regard to the angle formed between the long axis of the incisor and mandibular basal symphysis. The dentoalveolar symphysis includes the alveolar process and lower incisors. The long axis of the basal symphysis differs cephalometrically from the alveolar symphysis. However, they should be positioned as parallel to each other as possible and the overall angle created between them should not exceed 10° (Figure 6). Furthermore, the shape and the position of the basal symphysis cannot be influenced by the lower incisor tooth movement [8]. In case of a large angle, the inclination of the incisors is too big, requiring the repositioning of the teeth to correct the course of the long axis of the alveolar process and of the basal symphysis. The extent of this change is further determined by the increased reconstruction thickness in labial direction which allows the correct position of the teeth.

**Figure 7:**
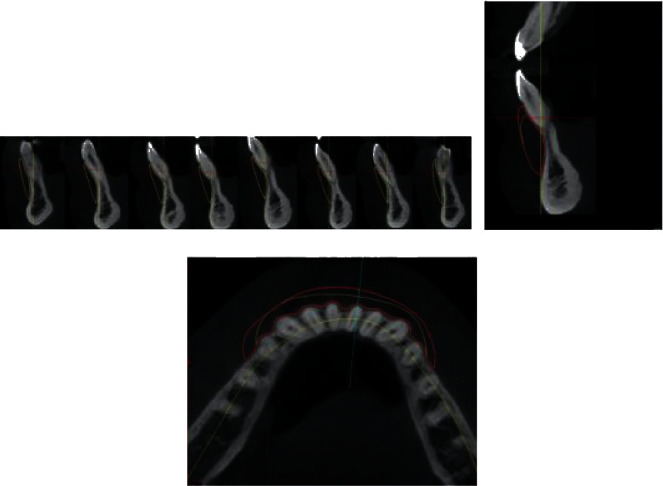
Planning of thickness of the bone block.

The other condition is the activity of the mental muscle which can be expressed by several parameters as the depth of the mentolabial sulcus, the “orange peel” symptom, and the measurement of muscle parameters in the cephalometric analysis (sections B–D) [19, 35, 36]. If the mental muscle shows high tension, the thickness of the graft resulting from bone measurements must not exceed the functional soft tissue envelope (Figure 7). If not eradicated, the excessive tension and activity can result in soft tissue fenestration as well as fenestration of the newly formed bone. The functional envelope should be evaluated in advance in cooperation with an osteopathic physician and/or treated with a topical application of botulinum toxin [37].

The bone shape and its position are also related to the occlusion. The occlusion, in turn, correlates with the axial position of the incisors. Again, the target values for the incisor position are taken from the analysis.

**Figure 8:**
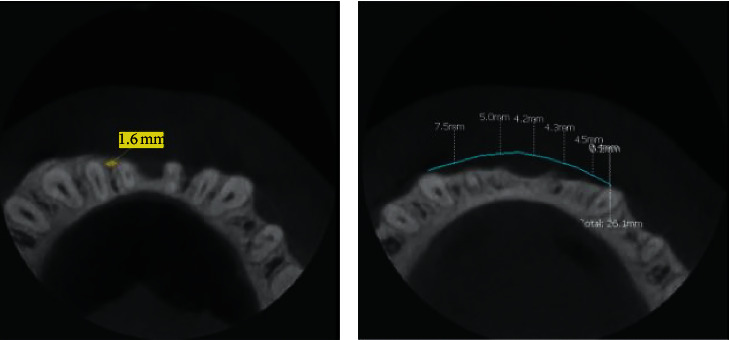
Shape of alveolar ridge in occlusal view. Measurements of interdental spaces width for positioning screws.

The implant width (regions 4a and 4b) depends on the size of the bone defect. The assessment can be carried out by two methods. The first method is subjective and is done on the basis of the analysis of the occlusal (horizontal) cross section of the alveolar process structure and shape. Under normal conditions, the lower jawbone together with its processes forms the shape of a horseshoe, with distinctly marked compact bone and cancellous bone of comparable widths on the right as well as on the left side. Lack of possibility to trace the parabola shape that corresponds to the curve of the digital program determines the scope of reconstruction (Figure 8). The second method is objective, using orthodontic analysis. The width is determined from the right to the left canine and must match the shape of the mandible in three dimensions. The width of the mandibular incisors determines the shape of the jaw. The incisor crown is measured, and the measured value determines the arch shape between the canine teeth. In the horizontal plane, the position of the teeth and alveolar process is drawn on CBCT scans and supplemented with the ideal size of the dental arch.

**Figure 9:**
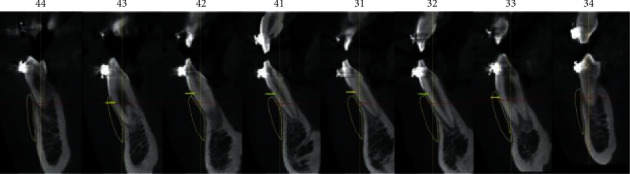
Estimation of proper range of bone block to the CEJ.

The height of the bone implant should be determined by 2 measurements—in the direction of the crown (region 1) and the apex (region 3). In the crown direction, periodontal parameters, including the biological width, should be taken into account (Figure 9). The level of the bones should be positioned at the CEJ level minus 2-3 mm, which corresponds to the biological width of each tooth. The discrepancy between 2 and 3 mm is due to the gingival biotype, which needs to be assessed prior to surgery. In case of a thin biotype, the width accounts for 2 mm, while in the thick biotype it is 3 mm. The biotype evaluation can be performed manually, using the puncture technique followed by reading the values from the periodontal millimeter scale or by ultrasound assessment [23]. In the chin area, the size of the implant is limited by muscle attachment, especially the insertion of the mental muscle. The chin position determines the range of the bone block. In the case of a strongly protruded chin and a receding alveolar ridge, the reference point is the largest protrusion of the chin. The retracted chin on the other hand can be reconstructed by changing its position by means of pushing it forward (pushing the Menton (Me) point forward). Thereafter, the scope of reconstruction becomes larger and the reference point is now the facial profile.

Next, the tooth movement is simulated by software such as Set-UP or ClinCheck®. While planning the therapy, the software allows individual steps to be discussed with the patient and the operating surgeons.

The necessary technical correction of ±1 mm in 3D is added to the target values. In this way, a correct size of bone is created for the movement of the tooth or implant.

#### 2.1.3. CAD/CAM

  Converting documents into digital models: based on CBCT and intraoral scanning, a virtual model of the mandible is created. A bone implant design is placed on this model. The respective actual and target value points are combined into one unit. The same applies to the merging of the four bone block divisions into one.
  Selection of suitable donor bones: before milling, the optimum size and shape of the bone block for processing should be selected. The necessary amount of cancellous and/or cortical bone can be taken from various parts of the human skeletal system—optimally, from areas presenting increased bone strain as the knees or hips. In those areas, the quantity and quality of bone beams are higher and additionally bone density is increased (D2-D3 according to Misch [38]), allowing the creation of a bone block which meets the correct strength criteria. It is important that the bone block is not fractured during the implementation procedure. Additionally, the usage of D4 type bone, which is too soft, may not effectively support the planned shape and size of the 3-dimensional structure under the influence of functional load of the masticatory muscles. This should especially be taken into account when it comes to large reconstructions and deep bone defects. The D4 bone makes it impossible to precisely prepare the alveolar concavities and convexities so that they fit very precisely into the interdental spaces. Exact linear contact, less than 0.4 mm, allows adequate transplant revascularization. It is known that the vascularization is obtained from 3 major sources—the periodontium, the spongy bone, and the periosteum. To improve the vascularization, decortication of the compact bone of the alveolar process should be performed in the surgical area. Still, the vascularization is limited by the reduced periodontium in advanced bone dehiscence or by periosteal incisions undertaken to mobilize a flap in incisal direction. As a result, the placement of growth factor concentrate in the form of an autogenous membrane between the alveolar process and the bone block is obligatory.

Due to the density of highly concentrated growth factors, including VEGF (vascular endothelial growth factor), neoangiogenesis is stimulated [39].

Additionally, an autogenous membrane consisting of growth factors is applied to the outer surface prior to closing the flap. Here, barrier membranes are not used due to the possible impairment of blood vessel penetration.

An exception is the apical region of the bone block, located close to the mental muscle.

In case of extended muscle mobilization, a natural collagen membrane covering a maximum of 1/3 of the bone block width in this area can be used. It limits the possible connective tissue growth under the bone block.

After milling, the block is cleaned, packed, and sterilised.

### 2.2. Bone Block Positioning Procedure

  Exact positioning of the bone block: after the preparation of the recipient bed, the bone block is placed on the alveolar ridge without excessive tension in a passive manner.
  The entire surface area as well as the different bone interfaces is smoothed out after the bone is placed in the recipient bed. Edges and irregularities are adjusted and rounded with the use of a special adapted diamond drill system to achieve a smooth junction between the bone and the implant. Remaining irregularities or gaps can be filled out with the use of allogenic bone particles. They can be placed directly under the bone block or on its edges.
  Positioning of the screws: the possible position of the anchor screws in the bone block is determined. When planning the placement of anchorage points in the bone implant, the risk of tooth damage and nerve damage needs to be excluded. The interdental spaces are the right position for placing the fixing screws. In the case of very narrow interdental spaces, titanium screws with a diameter of 1.2 mm should be used, and screws with a diameter of 1.6 mm (Figure 8) should be used for wider ones. Screws narrower than 1.2 mm should be avoided due to possible complications in removing them (e.g., screwhead breakage). The appropriate time for the screw removal is estimated to be 6 weeks after the procedure (bone healing time). The prolongation may cause the abovementioned complications. As an alternative, resorbable pins can be used; however, their prolonged resorption time which approximately takes up to one year and their large diameter of ca. 2 mm should be taken into consideration as this requires very wide interdental spaces.

The right screw position is planned in accordance with the axial distribution of the alveolar ridge load.

The screws must be placed in the middle of the bone block width, on both opposing sides—the place in front of the process/bone block fragment.

This allows greatest uniform pressure distribution on the bone block. For smaller blocks, 2 screws are used, while for larger ones, 4–6 screws are needed. The length of the screw depends on the thickness of the block and the alveolar process bone in the location where the screw is inserted. To avoid tunnelization, the apex of the screw should not penetrate the mucous membrane on the lingual side. This may result in complications, for example, the penetration of the soft tissue into the screw channel, leading to decreased screw anchorage and decreased bone block stabilization.

## 3. Summary

### 3.1. Results and Summary

Various bone augmentation techniques are known nowadays. Preventive bone augmentation prior to further therapy, e.g., orthodontic therapy or implantation, must form a unity with the existing bone. Only three-dimensional treatment planning gives us the possibility to obtain such results and in addition it gives us the possibility to move teeth into real bone after bone block implementation [39, 40]. The 3D technique should be used to prepare a fitting and precise bone block. It can be done in two ways: the first one, using printed jaw models, followed by the preparation of a bone block analogue. Usually a bone prototype is placed on the printed model to evaluate the exact range of the bone block; this implant must be tailored to the existing bone as a mismatched bone block can cause the irritation and inflammation of the surrounding soft tissue. Finally, the model is scanned and molded.

Disadvantage of this method is the possibility of making errors in the indirect measurements on the printed jaw models. The second technique is based on digital planning and molting carried out by the CAD/CAM system. In this case, the lack of indirect elements reduces the possibility of errors and precise bone block fitting is achieved. Furthermore, this technique reduces the surgical chair time and does not require manual adjustment.

The three-dimensional structure ensures stable support for the teeth and implant. The use of 3D technology ensures the best treatment results. By means of fully individualized bone, the technical problem of an imprecise fit is eliminated, and tooth regeneration is assured. If an incorrect augmentation material is chosen, tooth movement becomes difficult. The method ensures dimensional stability, which is particularly important in the mandible. This guarantees the creation of a new B-point and physiological bone remodelling.

Apart from one surgical site being involved and the simple surgical placement of the bone block, the procedure shows a positive effect on the patient's facial profile. The improved aesthetics of the facial profile is quickly noticed by the patient undergoing the treatment. All in all, this type of surgery harmonizes the facial proportions.

## Data Availability

The data are available in the Wroclaw Medical University data base and http://3ddent.eu/.
